# Endoplasmic reticulum stress response and transcriptional reprogramming

**DOI:** 10.3389/fgene.2014.00460

**Published:** 2015-01-07

**Authors:** Kezhong Zhang

**Affiliations:** Department of Immunology and Microbiology, Center for Molecular Medicine and Genetics, Karmanos Cancer Institute, Wayne State University School of MedicineDetroit, MI, USA

**Keywords:** ER stress, unfolded protein response, transcriptional reprogramming, metabolism, inflammation, oncogenes

Endoplasmic reticulum (ER) is an intracellular organelle responsible for protein folding and assembly, lipid and sterol biosynthesis, and calcium storage. As a protein folding compartment, the ER provides a high-fidelity quality control system to ensure that only correctly folded proteins are released from the ER and that unfolded and/or misfolded proteins are retained and degraded. A number of biochemical, physiological, or pathological stimuli can interrupt protein folding process, causing accumulation of unfolded or misfolded proteins in the ER lumen, a condition called “ER stress.” To cope with accumulation of unfolded or misfolded proteins, the ER has evolved a group of signaling pathways termed “Unfolded Protein Response (UPR)” or “ER stress response” that leads to transcriptional and translational reprogramming to either cope with the aberrant proteins or, under prolonged ER stress conditions, initiate programed cell death (Kaufman, 1999; Ron and Walter, 2007). To maintain ER homeostasis, transcriptional regulation mediated through multiple UPR branches is orchestrated to increase ER folding capacity, reduce ER workload, and promote degradation of misfolded proteins. The basic UPR pathways in mammalian cells consist of three main signaling cascades initiated by three ER-localized protein stress sensors: IRE1α (inositol-requiring 1α), PERK (double-strand RNA activated kinase-like ER kinase), and ATF6 (activating transcription factor 6). Relevant to transcriptional reprograming, the primary UPR transducer IRE1α functions as an RNase to splice the mRNA encoding X-box binding protein 1 (XBP1), which functions as a potent transcriptional activator that is functionally involved in immune response, cell metabolism, and cell survival. Alternatively, IER1α RNase can process select mRNA or pre-microRNA substrates, leading to degradation of mRNAs or microRNAs, a pathway was termed “Regulated IRE1-Dependent Decay (RIDD)” (Hollien and Weissman, 2006; Upton et al., 2012).

In recent years, accumulating evidence suggests that ER stress-triggered transcriptional reprogramming exists in many pathophysiological processes and plays fundamental roles in the initiation and progression of a variety of diseases, such as metabolic disease, cardiovascular disease, neurodegenerative disease, and cancer. Understanding effects and mechanisms of ER stress-associated transcriptional reprogramming has high impact on many areas of molecular genetics and is particularly informative to the development of pharmacologic avenues toward the prevention and treatment of modern common human diseases. To this direction, we have assembled a collection of timely research and review articles with a focus on ER stress response and transcriptional reprogramming in health and disease.

In this issue, Birk et al. contributed a method article describing the experimental framework to monitor stress-induced, time-resolved changes in ER reduction-oxidation (redox) states by using ER-targeted fluorescent biosensors (Birk et al., 2013). In the paper, advantages and drawbacks of existing techniques are discussed, and the power of these techniques was demonstrated in the context of selected cell culture models for ER stress. In regard to transcriptional reprogramming by ER stress response, a research paper by Arensdorf et al. described that the temporal clustering of gene expression links the metabolic transcription factor HNF4α to the ER stress-dependent gene regulatory network (Arensdorf et al., 2013a). In this work, they utilized a “bottom-up” approach to study the metabolic gene regulatory network controlled by the UPR in the liver, and provided a unique resource for the community to further explore the temporal regulation of gene expression during ER stress. Additionally, a review article from the same group discussed the non-canonical mechanisms and physiological consequences of the transcriptome induced by ER stress (Arensdorf et al., 2013b). As for non-canonical mechanism of stress-induced reprogramming, a review article by Coelho and Domingos summarized the physiological significance of transcriptional reprogram by RIDD, the ER stress pathway by which IRE1α processes and degrades mRNAs or pre-microRNAs, in several biological paradigms, including photoreceptor differentiation in Drosophila, mammalian liver, and endocrine pancreas function (Coelho and Domingos, 2014). They highlighted the importance of RIDD in tissues undergoing intense secretory function. Further, Horne et al. presented a newly emerging genome theory that unifies different types of stress and functional relationships from a genome-defined system point of view (Horne et al., 2014). They discussed the evolutionary relationship between stress and somatic cell adaptation under physiological, pathological, and somatic cell survival conditions, and advised to defocus from specific stresses and mechanisms by redirecting attention toward studying underlying general mechanisms.

In term of pathophysiological consequences of transcriptional reprogramming by ER stress response, an interesting research article by Han et al. described altered methylation and expression of ER-associated degradation (ERAD) factors in long-term alcohol-induced murine hepatic tumors (Han et al., 2013). Based on the data from ER chaperone BiP conditional knockout mice, they concluded that long-term alcohol consumption and aging may promote liver tumorigenesis in females through interfering with DNA methylation and expression of genes involved in the ERAD pathway. A review article by White-Gilbertson et al. discussed the current understanding of the involvement of ER stress response in multiple myeloma, an incurable plasma cell neoplasm cancer (White-Gilbertson et al., 2013). This paper highlighted that myeloma cells utilize ER stress response to gain unique metabolic signature, and therefore, inhibition of the ER stress response may represent a promising therapeutic strategy for multiple myeloma. As for metabolic disease, Zhou and Liu reviewed the current understanding of the involvement and mechanisms of ER stress response in dysregulation of hepatic lipid metabolism and hepatic lipotoxicity (Zhou and Liu, 2014). It is anticipated that understanding the roles and mechanism of ER stress response in hepatic lipid metabolism may lead to novel therapeutic strategies toward the control of metabolic disorders. Additionally, a review article from Guo and Li discussed the progress in understanding ER stress response in hepatic steatosis and inflammatory bowel diseases (Guo and Li, 2014). They highlighted the potential signaling pathways connecting ER stress with inflammation, and depicted the interplay between ER stress and inflammation in the pathogenesis of hepatic steatosis, inflammatory bowel diseases and colitis-associated colon cancer.

In summary, we anticipate that this collection of research and review articles from the front-running scientists will help the scientific community, clinical professionals, pharmaceutical industry, and public at large understand the mechanisms and pathophysiological significance of ER stress response. In particular, delineation of the molecular components of transcriptional reprogramming driven by ER stress response will shed new light on potential therapeutic strategies toward the control of modern common human diseases, such as metabolic disease, cardiovascular disease, immune disease, and cancer. While much work in ER stress-induced transcriptional reprogramming remains to be done, we hope that this topic will trigger more interest from the biomedical community in this important research direction.

## Conflict of interest statement

The author declares that the research was conducted in the absence of any commercial or financial relationships that could be construed as a potential conflict of interest.
